# Inequalities in geographic barriers and patient representation in lymphoma clinical trials across England

**DOI:** 10.1111/bjh.19907

**Published:** 2024-11-27

**Authors:** David A. Jones, Katie Spencer, Johanna Ramroth, Jake Probert, Laurence S. J. Roope, Rebecca Shakir, John Broggio, Frank Burroughs, Graham P. Collins, Philip M. Clarke, Jane L. Wolstenholme, David J. Cutter

**Affiliations:** ^1^ Nuffield Department of Population Health University of Oxford Oxford UK; ^2^ Leeds Institute of Health Sciences University of Leeds Leeds UK; ^3^ Oxford Cancer and Haematology Centre Oxford University Hospitals NHS Foundation Trust Oxford UK; ^4^ National Disease Registration Service NHS England Birmingham UK; ^5^ Bristol UK

**Keywords:** clinical trials, epidemiology, lymphomas

## Abstract

The distribution of trial site locations may lead to disparities in geographic access and affect patient representativeness in clinical trials. We utilised trial data covering 1993–2022 from the National Institute for Health and Care Research (NIHR) Open Data Platform, 2011 and 2021 English Census and geographic data and English individual‐patient cancer registry data for patients diagnosed with lymphoma between 1997 and 2017. To assess representation, we compared patient age and sex between trial participants and the incident population. We mapped the distance and travel times of English lower layer super output areas (LSOAs) to their nearest research active NHS Trusts and assessed associations between distance and travel times and the geographic and sociodemographic characteristics of the LSOAs. Trial participants were younger than the incident population and more likely to be male. The closest NHS Trust to more than half of English LSOAs was not research active. Greater LSOA mean age, male percent, White British percent, rurality and coastal/border status were positively associated with distance and travel time (at prespecified *p* < 0.05 level), while greater deprivation was negatively associated. Female and older lymphoma patients in England are underrepresented in trials, with the latter facing a higher burden of geographic barriers.

## INTRODUCTION

Despite a high willingness to participate in clinical trials, only a small proportion of patients typically enrol.^1^, ^2^, ^3^ From a patient's perspective, geographic barriers play a key role. Greater distance and travel time to a clinical trial site decreases desire and ability to participate.^4^, ^5^, ^6^ And more fundamentally, patients are typically treated at their most proximate healthcare centre which may not or may not be participating in a suitable trial. Notably, over half of patients with cancer report not participating due to a lack of available trials at their treating healthcare organisation.^7^

Although equality in access to research opportunities is a key tenet in the philosophies of various research funding bodies, the location of clinical trial sites reflects the needs and consideration of the trial organisers.^8^, ^9^ Investigators are under pressure to deliver research to time and budget which leads to the selection of sites based on professional networks, historical precedent and typical throughput of local patients at individual sites.^10^ This limits capacity to consider equality in access when planning research sites.^10^ In this study, we consider lymphoma clinical trial across England with the goal of assessing disparities in geographic barriers caused by the location of clinical trial sites and potential consequences in terms of social demographic inequalities and patient representation.

## METHODS

### Clinical trial identification and enrolled patient characteristics

Lymphoma‐specific interventional clinical trials in England were identified through the National Institute for Health and Care Research (NIHR) Open Data Platform (ODP).^11^ The NIHR ODP reports on clinical trials within the NIHR clinical research network (CRN) portfolio of trials in England. The search was performed in December 2022, with all indexed trials up to this date initially included. The characteristics of each clinical trial were obtained including opening and closing date, lymphoma subtype, study phase, line of treatment and age eligibility restrictions.

In England, NHS Trusts are responsible for the provision of secondary care and are typically the recruiting sites of interventional oncology trials. From 2009, information was available for each clinical trial on the participating NHS Trusts and the total number of patients recruited to the trial. From the financial year 2014–2015, the annual number of patients recruited at each participating NHS Trust for each clinical trial was also available. Permission to use and publish NIHR ODP data was granted by the NIHR and the Department of Health and Social Care.

For each clinical trial identified, we performed an extensive search for related publications to identify the characteristics of the enrolled patients and age eligibility restrictions. Only age and sex were extracted, as these were the only relevant patient characteristics reported.

### English lymphoma population data

Information on all individuals diagnosed with lymphoma, excluding Waldenström's macroglobulinaemia and small lymphocytic lymphoma, in England from 1997 to 2017 was obtained from the National Cancer Registration and Analysis Service (NCRAS). For each patient, we defined their NHS Trust of care as the Trust in which their treating team sits. This was primarily assigned according to the organisation recorded in the linked Cancer Waiting Time data as ‘the health care provider (NHS Trust) where the decision to treat the patient was made’. In a minority of patients, these data were missing. Here, we inferred a patient's NHS Trust of care from other organisational records in linked datasets (Data S1, Figure S1). NHS Trusts were defined as providing lymphoma care in England if they had at least one assigned patient.

As the organisation of NHS Trusts has changed slightly over time, we used the current naming configuration of NHS Trusts as of 2022 with mapping where the NHS Trust no longer existed. Finally, point of longitude and latitude were obtained for each 2022 NHS Trust to calculate distance and travel times.^12^

### English geographic data

To measure distance and travel times across England, we utilised small geographically defined area units of England called lower layer super output areas (LSOAs) which contain 1000–3000 people. Boundaries and population‐weighted centroids for each LSOA from the 2021 UK Census were obtained from the Office for National Statistics (ONS).^13^

Sociodemographic characteristics of each LSOA, based on the 2021 UK census, were also obtained from the ONS.^14^ This included population size, age, sex and ethnicity. Index of multiple deprivation (IMD) decile and rurality indicator were available only for LSOAs defined for the 2011 census.^15^, ^16^ These were mapped to their approximate 2021 LSOA.^17^ Finally, we created an indicator of whether the LSOA was either coastal or on the English border.^18^, ^19^ At present, there is no standard definition of LSOA coastal status; therefore, the indicator was based on the European Union definition of coastal areas: Local authority units that border the coastline or have at least 50% of their surface area within a distance of 10 km from the coastline.

### Study outcomes

We tabulated the characteristics of the identified studies including phase, randomisation status, line of treatment and total number of patients recruited. To assess representativeness, the extracted age and sex distributions of enrolled patients from each trial were pooled for each lymphoma subtype. Where participant age was reported in terms of median and (inter quartile) range, the mean and standard deviation were approximated using a common procedure.^20^ The combined mean age and sex distribution of the enrolled population was compared to that in the Cancer Registry data. Two tailed Welch's *t*‐test and chi‐squared test were used respectively. A *p*‐value of <0.05 was considered statistically significant.

The assessment of geographic barriers to trials requires the creation of one or more trial activity measures. The NIHR ODP data enable the use of the participation of NHS Trusts in trials and recruitment volume to construct such measures of NHS Trust trial activity, with subjective judgement required to determine how these are used to construct the measures. For this study, we chose to specify trial activity as a dichotomy, with an NHS Trust being classified as either ‘research active’ or ‘research inactive’ in five different ways according to the meeting of criteria as defined below.

For definition (1), the primary definition, NHS Trusts were dichotomised into being ‘research active’ or ‘research inactive’ according to the number of patients recruited into clinical trials. To account for the differing size of the catchment population treated by each NHS Trust, the total number of patients recruited by each NHS Trust was divided by the mean annual number of incident patients with lymphoma coming under their care. NHS Trusts were ordered by weighted recruitment, with the NHS Trusts who recruited above the median defined as ‘research active’.

Furthermore, as secondary definitions, noting the heterogeneity of lymphomas, we defined a ‘research active’ [and those not meeting the condition: ‘research inactive’] as; (2) an NHS Trust that participated in at least one lymphoma clinical trial, irrespective of lymphoma subtype; (3, 4 and 5) an NHS Trust that participated in at least one trial specifically in diffuse large B‐cell lymphoma (DLBCL), Hodgkin lymphoma (HL) and follicular lymphoma (FL) trial respectively.

The distance in kilometres from each LSOA population‐weighted centroid to the nearest NHS Trust that provided lymphoma care was calculated according to the haversine formula for the shortest distance between two points on a sphere. Additionally, the distance from each LSOA centroid to the nearest ‘research active’ NHS Trust was calculated.

To calculate travel times by car, we used the HERE REST application programming interface accessed through the R package *hereR*.^21^ Graphical mapping of distances and travel times by car were performed using the *R* package *tmap*.^22^ Multivariable linear regression was used to assess associations between the distance of each LSOA to their nearest ‘research active’ NHS Trust with LSOA sociodemographic characteristics, namely, mean age, sex, ethnicity, IMD decile, rurality and coastal status.

To measure geographic‐associated knowledge barriers to access, we calculated the proportion of the total English mean annual incident population treated in ‘research active’ and ‘inactive’ NHS Trusts, according to the five definitions. Here, the mean annual incidence was calculated using patients diagnosed between 2014 and 2017 in the English Cancer Registry data. Furthermore, we compared the mean age, sex proportion, White British ethnicity proportion and IMD income domain quintile characteristics of Cancer Registry patients treated in ‘research active’ NHS Trusts against those treated in ‘research inactive’ NHS Trusts. Two‐tailed Welch's *t*‐test and chi‐squared test, including test for trend for IMD quintile, were again utilised where appropriate.

## RESULTS

### Lymphoma‐specific clinical studies

We identified 76 interventional clinical trials in lymphoma recorded in the NIHR ODP, and a complete table of studies is provided in Data S2. The earliest study recorded in the NIHR ODP opened in 1993, and the latest, after exclusion for study status, opened in 2021. Most of the identified clinical trials were phase II and phase III, with an almost equal proportion randomised and non‐randomised. A full tabular break down of the characteristics of the clinical trials is given in Data S1.

### Variation in study activity

**FIGURE 1:**
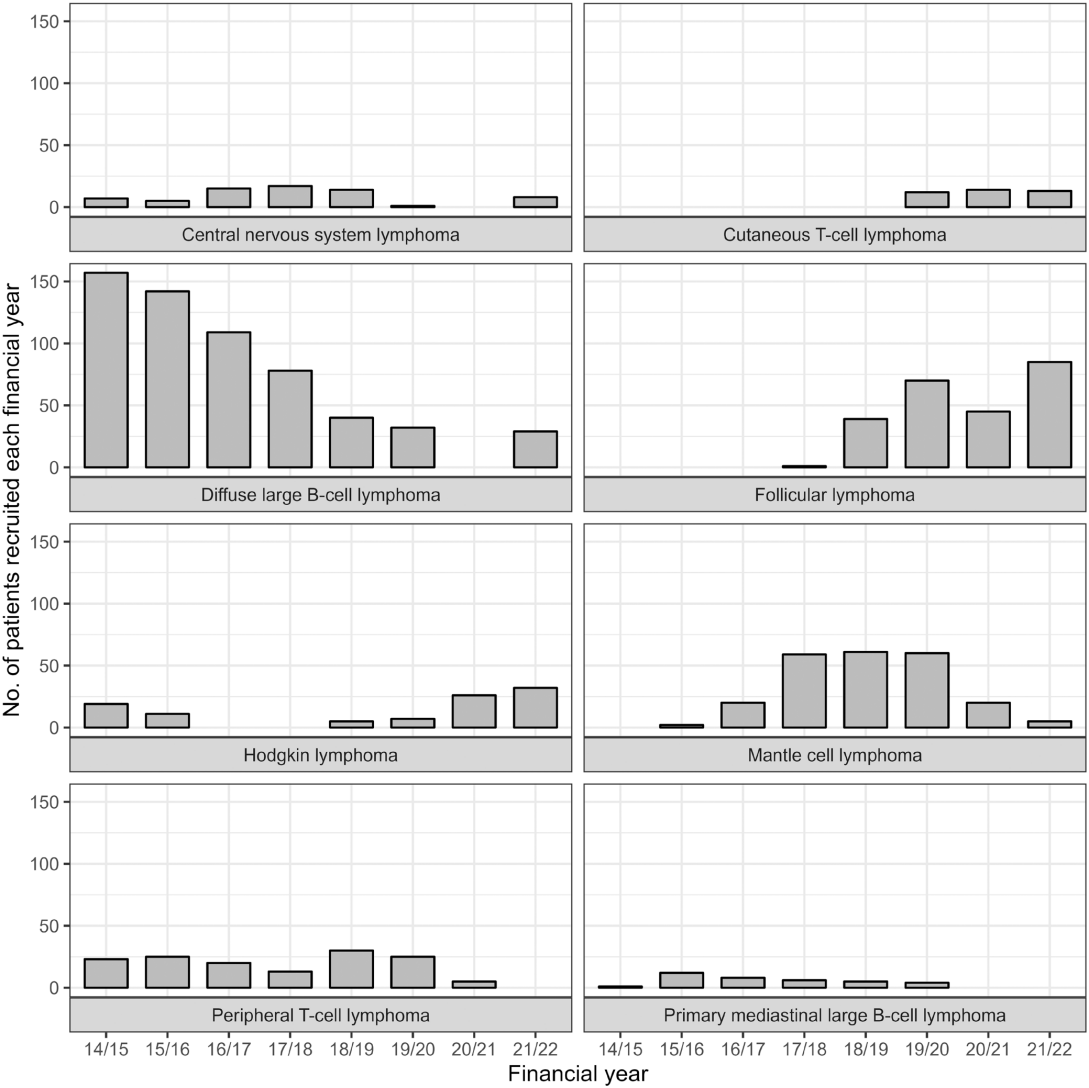
Number of patients recruited each year by lymphoma diagnostic subtype.

Using the NIHR ODP annual recruitment data from 2014/15 to 2021/22, we found temporal variation in the recruitment of patients to clinical trials for each lymphoma subtype (Figure 1). There were five lymphoma subtypes (DLBCL, HL, mantle cell lymphoma [MCL], peripheral T‐cell lymphoma [PTCL] and Primary mediastinal large B‐cell lymphoma (PMBCL)) with a first‐line clinical trial open to enrolment in the years from 2014/15 to 2021/22, and therefore having more granular recruitment data within the NIHR ODP. The estimated ratio of the mean annual number patients enrolled over the mean annual number diagnosed suggests approximately 1/100 newly diagnosed patients with DLBCL, 1/200 newly diagnosed patients with HL, 1/20 newly diagnosed patients with MCL, 1/100 newly diagnosed patients with PTCL and patients with 1/10 newly diagnosed PMBCL participate in a first‐line clinical trial.

### NHS trust clinical trial activity

There were 127 NHS Trusts identified as providing care for patients with lymphoma from the NCRAS data. Of these 110 (87%) NHS Trusts were identified in the NIHR ODP as having participated in a lymphoma trial since 2009, including those that did not recruit any patients. The average number of patients recruited at these NHS Trusts was 36 patients, with a range from 0 to 252. Using the annual recruitment data available from the financial year 2014/15 onwards, there were 80 (63%) NHS Trusts that recruited one or more patients to a clinical trial between then and 2021/22.

**FIGURE 2:**
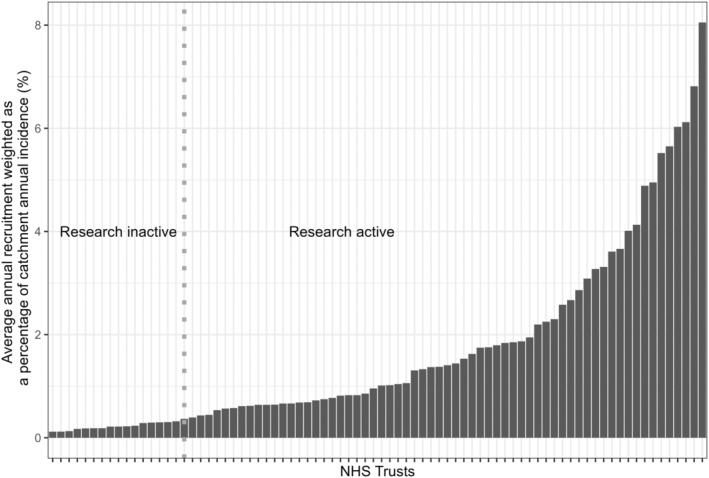
Mean annual percentage of lymphoma patients recruited by each positively recruiting NHS Trust weighted by the size of their catchment annual incident population.

In Figure 2, the average annual recruitment for the 80 NHS Trusts, weighted by the size of their annual incident population, is presented. Average annual recruitment at each NHS Trust was calculated from the NIHR ODP annual recruitment data for the financial year (FY) from 2014/15 to 2021/22 and annual incident population calculated from the cancer registry data based on diagnoses between the years 2014 and 2017. Definition (1) of research activity is based on the midpoint of the 127 NHS Trusts ordered by this weighted recruitment, that is, the 80 NHS Trusts with positive recruitment and the 47 without. Under this definition, 63 NHS Trusts were considered ‘research active’. Under the less restrictive definition (2) of research activity, whether an NHS Trust recruited one or more patients to any trial, 96 NHS Trusts were considered ‘research active’. Specifically looking at recruitment to DLBCL, HL and FL trials, 70, 17 and 29 NHS Trusts, respectively, were considered ‘research active’.

### Representativeness of participating patients

Published full articles and conference abstracts were searched for all 76 clinical trials. Publications were identified for 57 of the trials. Of the 19 without publications, six were completed trials, 11 were still open to recruitment or in follow‐up, one trial did not recruit any patients in England, and one trial was terminated without publication. Summary age and sex statistics of enrolled patients were routinely reported in almost all publications, but no other patient‐specific information was commonly reported.

Across the specified lymphoma subtypes, most enrolled patients were male, and the male proportion was consistently higher than that of the total English lymphoma population. This difference in sex proportion was statistically significant for DLBCL, FL, MCL and PTCL, where the percentage of males recruited was 2.3%, 2.7%, 5.6% and 6.1% higher than the percentage of male patients in the total subtype population. Full results are provided in Data S1.

The mean age of participants was consistently lower than that of the mean age at diagnosis of the population. For some subtypes, the absolute difference was substantial with patients recruited to DLBCL, Burkitt lymphoma and marginal zone lymphoma being on average 10.9, 11.9 and 10.5 years younger than the average age of the total respective subtype populations. Restricting the age comparison to include first‐line trials only, such that age of enrolment more closely reflected age of diagnosis, had little impact on combined mean age and standard deviation (Data S2). Neither did the results change when restricting the age comparison to trials with no age limiting eligibility criteria (Data S2). Restricting the comparison to phase II trials only increased mean age of participants compared to the first line and age limit scenarios; however, mean age, with the exception of Hodgkin lymphoma, remained lower than that of the total subtype populations.

### Distance and travel time to ‘research active’ providers

**FIGURE 3:**
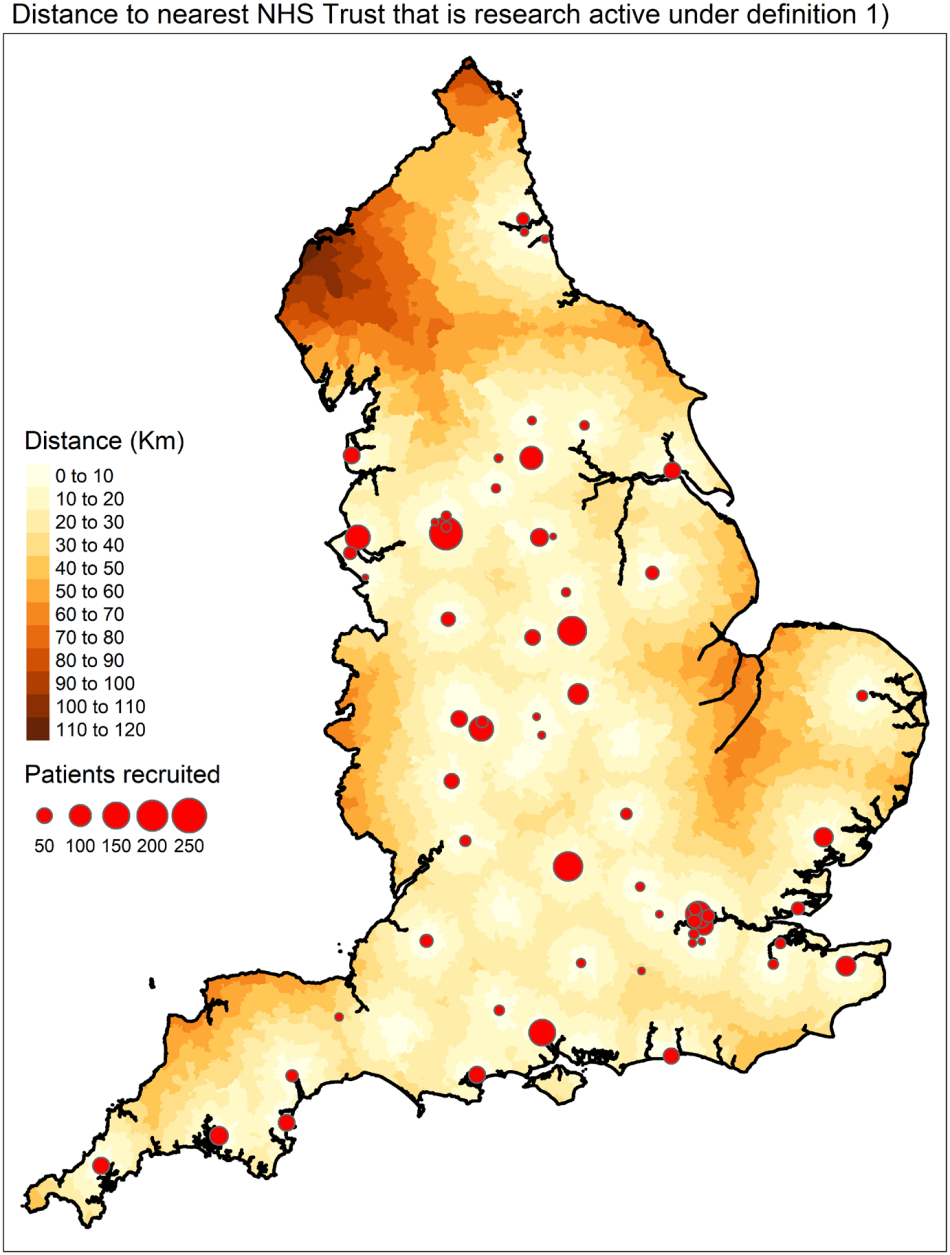
Distance from each LSOA to nearest ‘research active’ NHS Trust under definition (1): An NHS Trust that recruited more than the median of average annual trust recruitment weighted by annual trust incidence. LSOA, layer super output area.

The median distance (travel time) from the centroid of each LSOA to the nearest ‘research active’ NHS Trust, under definition (1) of research activity was 15.6 km (22.1 min) with an interquartile range of 5.5–21.6 km (12.7–28.1 min) and maximum of 115.7 km (97.1 min). Figure 3 maps this distribution of distances across England. Analogous maps for the four other definitions are provided in Data S1 (Figures S2–S5). Average distances (travel times) for all definitions of research activity are given in Data S1. Distances and travel times increased greatly when focusing on the HL and FL ‘research active’ centres.

**FIGURE 4:**
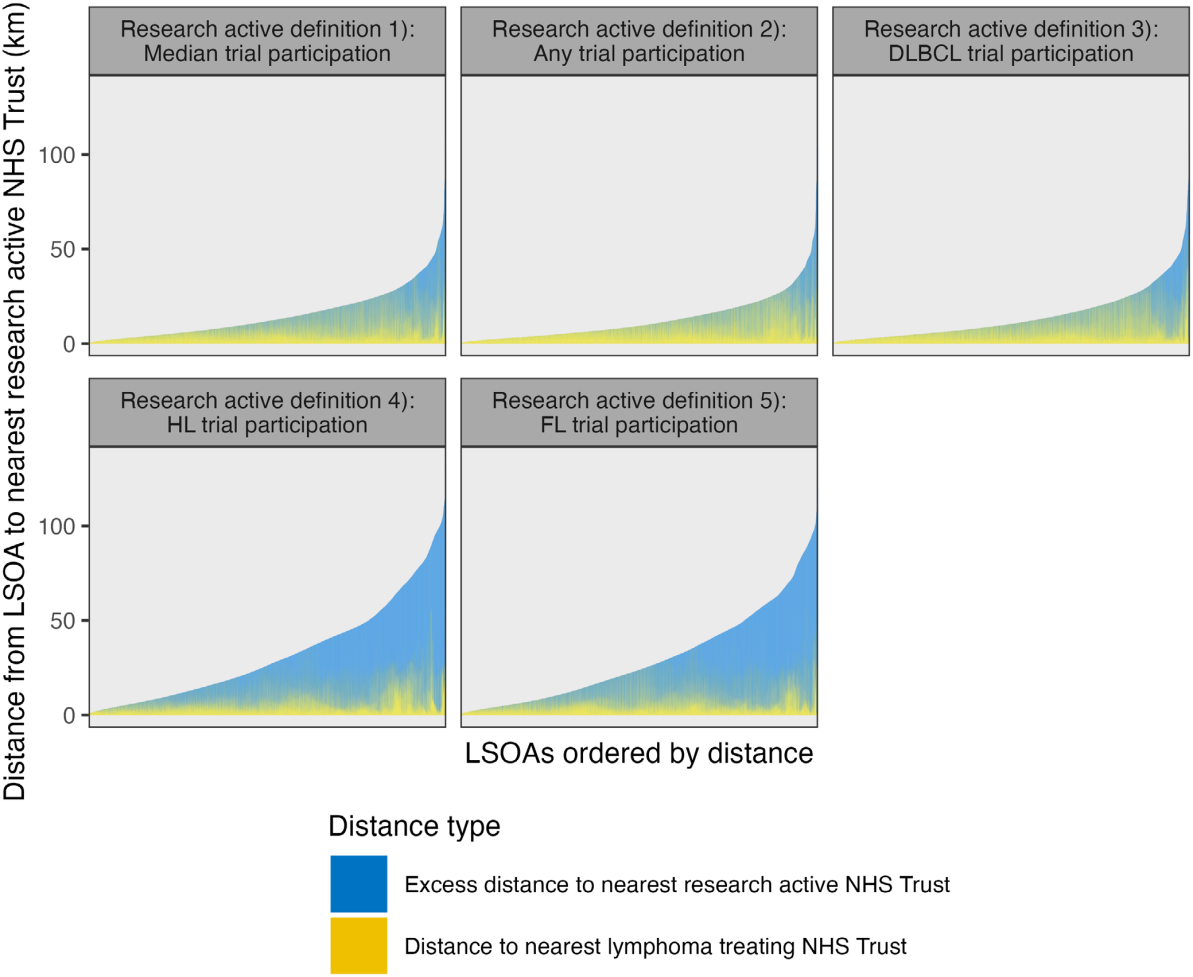
Distribution of total travel times to nearest ‘research active’ NHS Trust under definition (1), including excess distance where the ‘research active’ NHS Trust is not the closes lymphoma treating NHS Trust. DLBCL, diffuse large B‐cell lymphoma; LSOA, layer super output area.

For nearly half (46.5%) of all LSOAs, their nearest lymphoma treating NHS Trust was not ‘research active’ under definition (1). This increased to 75% and 84% when using the FL and HL trials definitions, respectively. Figure 4 graphs the distribution of total distance from each LSOA to the nearest ‘research active’ NHS Trust for all five ‘research active’ definitions. This includes the excess distance where their nearest ‘research active’ NHS is not their nearest lymphoma treating NHS trust.

Results from the linear regression analyses of LSOA characteristics on distance to nearest ‘research active’ NHS Trust, according to definition (1), are given in Table 1. Results for the other definitions are given in Data S1. Greater distances were positively associated with LSOA mean age, percentage male and percentage White British ethnicity, rural status and coastal/border status. There was a negative association with IMD decile, with more deprived areas having less distance/time to travel.

**TABLE 1:** Multivariable linear regression regarding the association between LSOA characteristics and distance (km) to nearest ‘research active’ NHS Trust.

LSOA characteristics	Coefficient	Standard error	*p*‐Value
Mean age	0.400	0.022	<0.001
Percentage male	0.386	0.035	<0.001
Percentage White British	0.123	0.004	<0.001
IMD decile	−0.216	0.028	<0.001
Rural status	8.003	0.207	<0.001
Coastal/border status	2.750	0.225	<0.001
Constant	−22.189	2.036	<0.001

Abbreviations: IMD, index of multiple deprivation; LSOA, lower layer super output area.

### NHS Trust research activity and patient characteristics

Utilising again our assignment of patients in the English Cancer Registry to their NHS Trust of care, and the resulting mean annual number of patients diagnosed over the years 2014–2017 from the English cancer registry, we calculated the relative proportion of lymphoma patients, by subtype, cared for by each NHS Trust. For DLCBL, FL and HL, 66.7%, 25.1% and 32.1% of patients, respectively, were treated in ‘research active’ NHS trusts using the subtype specific definition.

Table 2 compares the characteristics of patients with DLBCL, HL and FL between DLBCL/HL/FL ‘research active’ and ‘inactive’ NHS Trusts. For all three diseases, there was a statistically significant difference in mean age given by their respective two‐tailed Welch's *t*‐tests. The magnitude of the difference was most marked for HL, with the mean age of patients under the care of ‘research active’ NHS Trusts 5.6 years less than that in ‘research inactive’ NHS Trusts. Elsewhere, there was also a statistically significant difference in the distribution of patient IMD income domain quintile.

**TABLE 2:** Comparison of cancer registry patient characteristics between ‘research active’ and ‘inactive’ NHS Trusts.

Disease		‘Research active’ NHS Trusts	‘Research inactive’ NHS Trusts	*p* Value
DLBCL	No. patients	19 769	9809	‐
	Age: Mean (SD)	67.8 (14.6)	69.1 (13.9)	<0.001
	Males (%)	55%	55.4%	0.504
	White British ethnicity (%)	87.4%	87.3%	0.867
	IMD income domain: % in two most deprived quintiles	34.1%	34.5%	0.304
HL	No. patients	2419	7166	‐
	Age: Mean (SD)	43.6 (19.9)	49.2 (20.7)	<0.001
	Males (%)	54.9%	56.2%	0.272
	White British ethnicity (%)	77.9%	80.9%	0.001
	IMD income domain: % in two most deprived quintiles	44.2%	39.2%	<0.001
FL	No. patients	4682	9324	‐
	Age: Mean (SD)	64.5 (13.6)	65.0 (13.1)	0.041
	Males (%)	47.4%	48.8%	0.130
	White British ethnicity (%)	87.9%	88.4%	0.346
	IMD income domain: % in two most deprived quintiles	33.3%	31.9%	0.115

Abbreviations: DLBCL, diffuse large B‐cell lymphoma; FL, follicular lymphoma; HL, Hodgkin lymphoma; IMD, index of multiple deprivation; SD, standard deviation.

## DISCUSSION

Our analysis identified underrepresentation of older and female participants in lymphoma clinical trials. These results align with a recent study of representation in pivotal US clinical trials in lymphomas.^23^ Furthermore, our findings demonstrate that the current (and historically precedent) location of trial sites causes significant disparities in geographic barriers across England. Coastal and rural LSOAs face the largest distances, and greater distance was associated with higher area mean age. For most LSOAs in England, their nearest NHS Trust is not ‘research active’. Most patients in these LSOAs would therefore be required to change the NHS Trust they are treated at to access the trial.

We consistently found that, even in trials with no explicit upper age restriction, older patients are underrepresented when considering the mean age of participants. Coupled with our other results, this finding may relate to the geographical availability of ‘research active’ hospitals. Increased distance/travel time has a well‐studied deleterious effect on participation.^24^, ^25^, ^26^ And a lack of trial availability at a patient's treating centre is one of the most significant barriers to access.^7^ This relates both to a lack of knowledge of available trials and also the need to change hospital and treating doctor in order to participate which has a compounding effect on willingness to travel.^6^ Only a few of the trial studies supplied results on age characteristic of participants beyond the mean or median, precluding analysis assessing whether representation differed across age strata. Further reporting of patient characteristics in trial publication would aid in future assessment of disparities.

The underrepresentation of females did not correlate with our geographic access measures. Such underrepresentation has been widely found across trials for many diseases, although England specific analyses relating to oncology trials are lacking.^27^, ^28^ This could relate in part to age effects, with women having higher relative incidence in the very highest age categories.^29^ Additionally, women appear to exhibit a lower willingness to participate than men, which may relate to intrinsic risk preferences.^30^, ^31^ And women are less likely to participate in studies led by men, perhaps amplifying trial participation disparities in cancer trials when viewed alongside current gender inequity in cancer research leadership.^32^, ^33^, ^34^, ^35^

A limitation in using the NIHR ODP is that it contains only those trials receiving support from the NIHR CRN. Some industry‐sponsored phase I studies will have been missed consequently, but these studies tend to recruit a relatively small number of patients with relapsed disease and limited conventional treatment options, so their inclusion would not substantially affect the conclusion of this study. Further limitations of our study include the need to subjectively define and categorise research activity, which may not reflect temporal changes over our 20‐year timeframe. Our measures of geographic access capture proximity and travel time by car; however, many patients will not have access to a car or be fit to drive, greatly increasing the time and travel burden to their nearest centre. Those more deprived for instance have far lower car ownership.^36^ Finally, although we bring together disparate datasets on trial recruitment and individual patient cancer registry data, which intersect at the NHS trust level, our analysis is constrained by a lack of individual‐patient linkage. Future work, given the availability of patient linkages between trial and cancer registries, may look to assess the revealed effects of distance, travel times and catchment, which may be heterogeneous across sociodemographic characteristics.

Reducing inequalities in access aligns with the principles of many research funders and features significantly in the United Kingdom's recent policy paper on the future of clinical research delivery and the independent Lord O'Shaughnessy review on commercial clinical trials in the United Kingdom.^8^, ^9^, ^37^ Our study suggests that, for lymphoma, female and older patients are underrepresented in trials for most lymphoma subtypes, with the latter facing a greater higher burden of geographic barriers.

## AUTHOR CONTRIBUTIONS

DAJ, DJC, PMC and JLW conceptualised the study. JR and JP processed the individual‐level patient data received from the National Disease Registration Service. DAJ performed the analysis and drafted the manuscript. All authors critically reviewed the manuscript and approved the final submitted version.

## FUNDING INFORMATION

DAJ was funded through a Nuffield Department of Population Health DPhil scholarship. DJC, JR and JP were funded by Cancer Research UK (grant number C8225/A21133 and PRCRPG‐Nov21/100001). RS was funded by a National Institute of Health Research Doctoral Fellowship (grant number NIHR300740). LSJR and PMC are supported by the National Institute for Health Research Oxford Biomedical Research Centre.

## CONFLICT OF INTEREST STATEMENT

DAJ is an employee of GRAIL Bio UK. GRAIL Bio UK had no role in the conception, design, analysis or submission of this study.

## ETHICS APPROVAL STATEMENT

The study was approved by the National Cancer Registration and Analysis Service's Office for Data Release (reference ODR1920_011). The study makes use of routinely collected data that was de‐personalised before release.

## CONSENT TO PARTICIPATE

Informed consent from individual participants was therefore not required.

## Supporting information


Data S1.

Data S2.

## Data Availability

Data on clinical trial recruitment are freely accessed using the NIHR Open Data Platform (https://odp.nihr.ac.uk). The cancer registry data analysed for this paper are securely held by the National Disease Registration Service (NDRS). Requests to access the data can be made through NHS England's DARS service (https://digital.nhs.uk/services/data‐access‐request‐service‐dars). Analytical code will be provided by the corresponding author on request.
