# Supplementary material for: Inequalities in geographic barriers and patient representation in lymphoma clinical trials across England
Source: Br J Haematol. 2024 Nov 27;206(2):531–40. doi: 10.1111/bjh.19907 (PMC11829142; doi:10.1111/bjh.19907)

**Supplementary Information**

**Supplemental Tables**

**Table S1: Characteristics of the 76 identified interventional clinical trials in lymphoma**

|  | Categories | No. (%) or ratio |
| --- | --- | --- |
| Phase | I | 5 (7%) |
|  | I/II | 4 (5%) |
|  | II | 34 (45%) |
|  | II/III | 2 (3%) |
|  | III | 29 (38%) |
|  | NA | 2 (3%) |
| Randomised | Randomised | 41 (54%) |
|  | Non-randomised | 34 (45%) |
|  | Both | 1 (1%) |
| Lymphoma subtype | Diffuse large B-cell | 14 (18%) |
|  | Hodgkin | 12 (16%) |
|  | Follicular | 10 (13%) |
|  | Mantle cell | 7 (9%) |
|  | Peripheral T-cell | 6 (8%) |
|  | Primary CNS | 5 (7%) |
|  | Waldenström’s macroglobulinaemia | 4 (5%) |
|  | Cutaneous T-cell | 3 (4%) |
|  | Burkitt | 2 (3%) |
|  | Primary mediastinal large B-cell | 2 (3%) |
|  | Marginal zone | 1 (1%) |
|  | Other/mixtures | 10 (13%) |
| Treatment line | First line | 42 (55%) |
|  | Relapse/refractory | 30 (39%) |
|  | Other | 4 (5%) |
| Opening year | 1993 - 2000 | 8 (11%) |
|  | 2001 - 2005 | 13 (17%) |
|  | 2006 - 2010 | 22 (29%) |
|  | 2011 - 2015 | 16 (21%) |
|  | 2015 - 2022 | 17 (22%) |
| Total no. of English patients enrolled | 0 - 50 | 42 (55%) |
|  | 51 - 100 | 9 (12%) |
|  | 101 - 200 | 7 (9%) |
|  | 200+ | 11 (14%) |
|  | Open to recruitment | 7 (9%) |
| Age eligibility restrictions | No information | 5/76 |
|  | Lower age restriction of 16 years | 18/72 |
|  | Lower age restriction of 18 years | 49/72 |
|  | Lower age restriction above 18 years | 4/72 |
|  | Any upper age restriction | 4/72 |

**Table S2: Comparison of mean age and sex ratio between enrolled patients and incident population**

|  | Clinical trials (1993 - 2021) | |  | | | English population (1997 - 2017) | | | |  | | P value | |
| --- | --- | --- | --- | --- | --- | --- | --- | --- | --- | --- | --- | --- | --- |
|  | Total No. of patients enrolled; sex (age) | Prop. male | | Age at enrolment; Mean, SD |  | Total No. of patients | Prop. male | | Age at diagnosis; Mean, SD | |  | Sex prop. | age |
| Diffuse large B-cell | 1757 (2957) | 0.567 | | 56.2 (12.3) |  | - | - | - | |  | | - | - |
| CNS (DLBCL) | 334 (334) | 0.593 | | 57.0 (9.5) |  | - | - | - | |  | |  |  |
| Combined | 2091 (3291) | 0.571 | | 56.3 (12.0) |  | 65440 | 0.548 | 67.2 (14.7) | |  | | 0.039 | < .0001 |
| Hodgkin | 3385 (3416) | 0.585 | | 41.7 (13.4) |  | 27319 | 0.571 | 46.6 (19.8) | |  | | 0.129 | < .0001 |
| Follicular | 3005 (3005) | 0.506 | | 57.5 (11.2) |  | 33911 | 0.479 | 63.6 (13.6) | |  | | < .001 | < .0001 |
| Mantle | 612 (612) | 0.766 | | 63.1 (9.6) |  | 5219 | 0.710 | 70.0 (11.8) | |  | | < .01 | < .0001 |
| Peripheral T-cell | 213 (231) | 0.718 | | 60.1 (12.8) |  | 7061 | 0.600 | 63.7 (16.1) | |  | | < .0001 | < .0001 |
| Cutaneous T-cell | 57 (57) | 0.667 | | 63.0 (11.5) |  | 6230 | 0.606 | 62.2 (16.3) | |  | | 0.425 | 0.609 |
| Burkitt | 58 (58) | 0.793 | | 41.8 (12.8) |  | 2101 | 0.707 | 53.7 (19.0) | |  | | 0.200 | < .0001 |
| Primary mediastinal large B-cell | 125 (125) | 0.384 | | 33.7 (10.5) |  | 360 | 0.397 | 41.5 (16.8) | |  | | 0.877 | < .0001 |
| Marginal zone | 401 (401) | 0.491 | | 57.3 (9.0) |  | 12782 | 0.476 | 67.8 (13.6) | |  | | 0.576 | < .0001 |

**Table S3: Mean and interquartile range of the distance and car travel time to nearest ‘research active’ NHS Trust by the research activity definitions**

| Research active definitions | Mean distance (car travel time) | Interquartile range |
| --- | --- | --- |
| 1) An NHS Trust that recruited more than the median of average annual Trust recruitment weighted by annual Trust incidence. | 15.6km (22.1mins) | 5.5 – 21.6km (12.7 – 28.1mins) |
| 2) An NHS Trust that participated in at least one lymphoma clinical trial | 13.2km (19.3mins) | 4.5 – 18.2km (11.4 – 24.6mins) |
| 3) An NHS Trust that participated in at least one DLBCL trial | 14.7km (21.0mins) | 5.1 – 20.2km (12.2 – 26.6mins) |
| 4) An NHS Trust that participated in at least one HL trial | 32.6km (40.1mins) | 11.2 – 46.6km (21.9 – 51.5mins) |
| 5) An NHS Trust that participated in at least one FL trial | 30.2km (39.1mins) | 9.4 – 45.1km (18.9 – 52.6mins) |

**Table S4: Coefficients, standard error, and P-values from the OLS regression of LSOA characteristics on the distance to their nearest research active NHS Trust under definition 2): an NHS Trust which participated in any trial.**

| LSOA characteristics | Coefficient | Standard error | P value |
| --- | --- | --- | --- |
| Mean age | 0.47207 | 0.01997 | <0.001 |
| Percentage male | 0.33268 | 0.03140 | <0.001 |
| Percentage white British | 0.11317 | 0.00404 | <0.001 |
| IMD decile | 0.29459 | 0.02469 | <0.001 |
| Rural status | 7.73630 | 0.18428 | <0.001 |
| Coastal/border status | 2.79718 | 0.20105 | <0.001 |
| Constant | -23.95907 | 1.81294 | <0.001 |

**Table S5: Coefficients, standard error, and P-values from the OLS regression of LSOA characteristics on the distance to their nearest research active NHS Trust under definition 3): an NHS Trust which participated in any DLBCL trial.**

| LSOA characteristics | Coefficient | Standard error | P value |
| --- | --- | --- | --- |
| Mean age | 0.442271 | 0.022177 | <0.001 |
| Percentage male | 0.326540 | 0.034876 | <0.001 |
| Percentage white British | 0.133320 | 0.004487 | <0.001 |
| IMD decile | -0.277210 | 0.027421 | <0.001 |
| Rural status | -8.311566 | 0.204701 | <0.001 |
| Coastal/border status | 2.009902 | 0.223326 | <0.001 |
| Constant | -22.090921 | 2.013801 | <0.001 |

**Table S6: Coefficients, standard error, and P-values from the OLS regression of LSOA characteristics on the distance to their nearest research active NHS Trust under definition 4): an NHS Trust which participated in any HL trial.**

| LSOA characteristics | Coefficient | Standard error | P value |
| --- | --- | --- | --- |
| Mean age | 0.182938 | 0.041025 | <0.001 |
| Percentage male | 0.903412 | 0.064516 | <0.001 |
| Percentage white British | 0.352950 | 0.008301 | <0.001 |
| IMD decile | 0.073756 | 0.073756 | 0.146 |
| Rural status | 10.860964 | 0.378672 | <0.001 |
| Coastal/border status | 17.677702 | 0.413127 | <0.001 |
| Constant | -41.716139 | 3.725295 | <0.001 |

**Table S7: Coefficients, standard error, and P-values from the OLS regression of LSOA characteristics on the distance to their nearest research active NHS Trust under definition 5): an NHS Trust which participated in any FL trial.**

| LSOA characteristics | Coefficient | Standard error | P value |
| --- | --- | --- | --- |
| Mean age | 0.59612 | 0.04171 | <0.001 |
| Percentage male | 0.92193 | 0.06560 | <0.001 |
| Percentage white British | 0.20641 | 0.00844 | <0.001 |
| IMD decile | -0.52459 | 0.05158 | <0.001 |
| Rural status | 9.12268 | 0.38503 | <0.001 |
| Coastal/border status | 0.78673 | 0.42006 | <0.001 |
| Constant | -45.84592 | 3.78783 | 0.006 |

**Supplemental Figures**

**Figure S1: Decision algorithm to allocate NHS Trust of care based on the missingness of variables from obtained NCRAS datasets.**

**
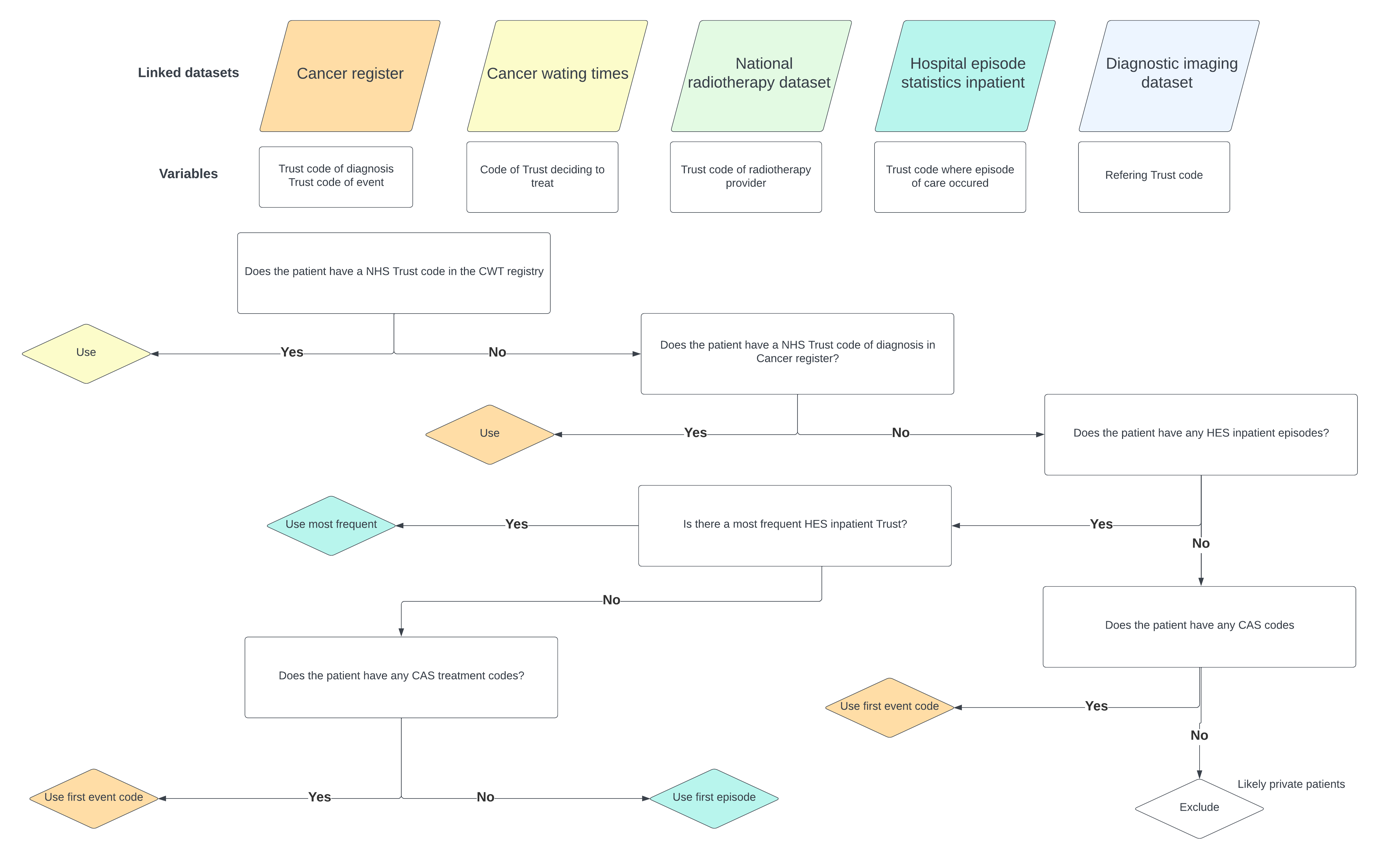
**

**Figures S2: Distance from each LSOA to nearest research active NHS Trust under definition 2): an NHS Trust that recruited to any clinical trial.**

**
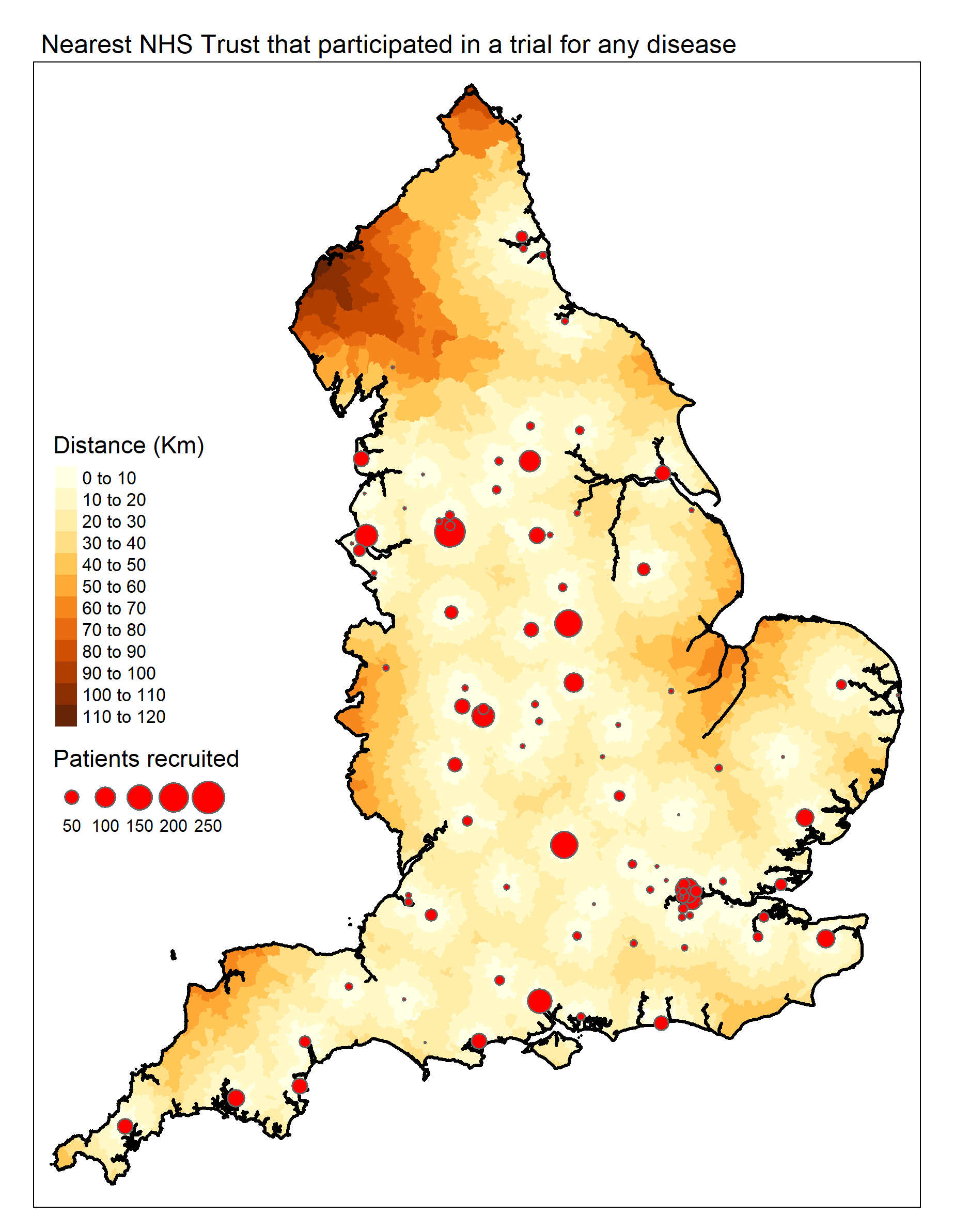
**

**Figures S3: Distance from each LSOA to nearest research active NHS Trust under definition 3): an NHS Trust that recruited to any DLBCL clinical trial.**

**
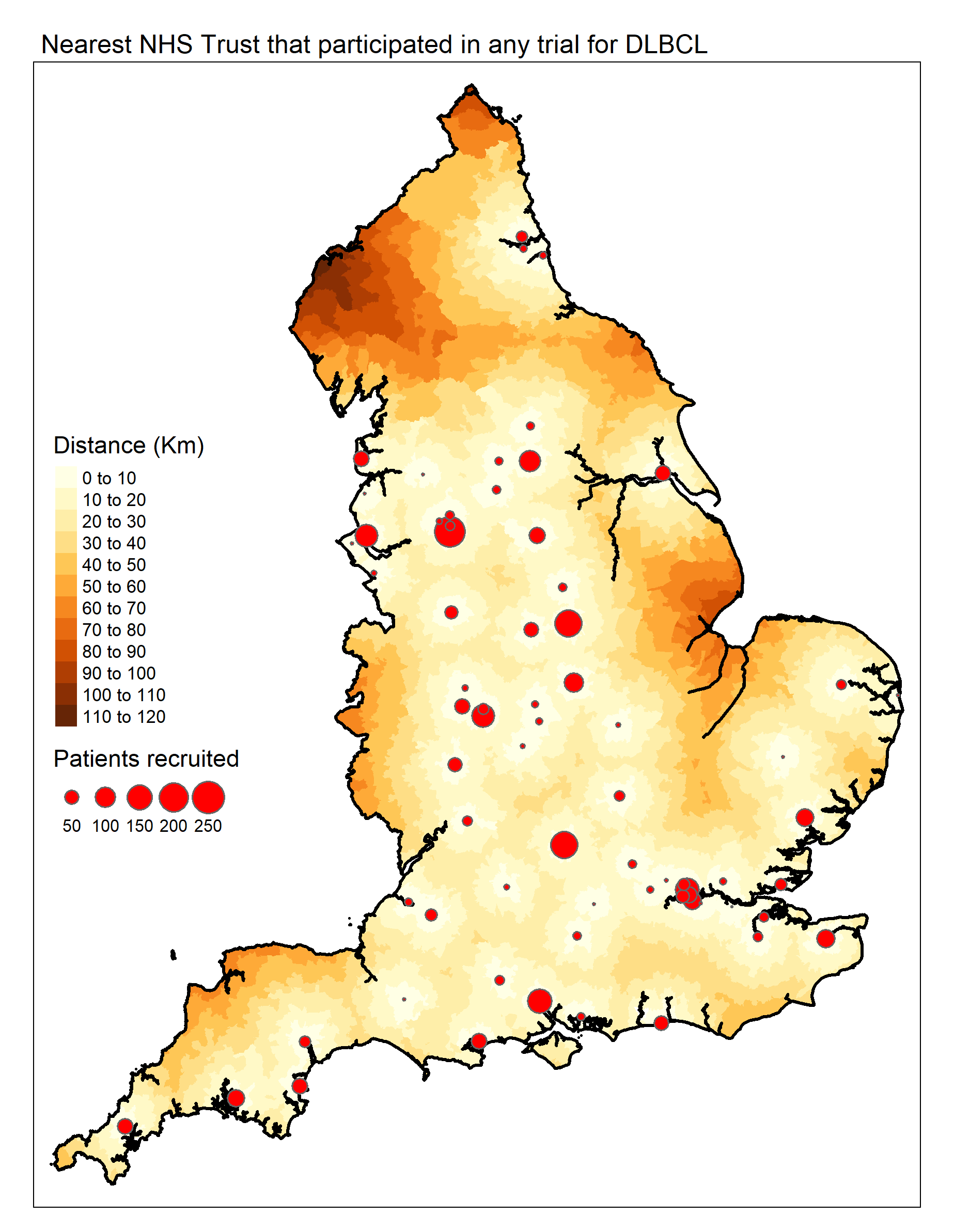
**

**Figures S4: Distance from each LSOA to nearest research active NHS Trust under definition 4): an NHS Trust that recruited to any HL clinical trial.**

**
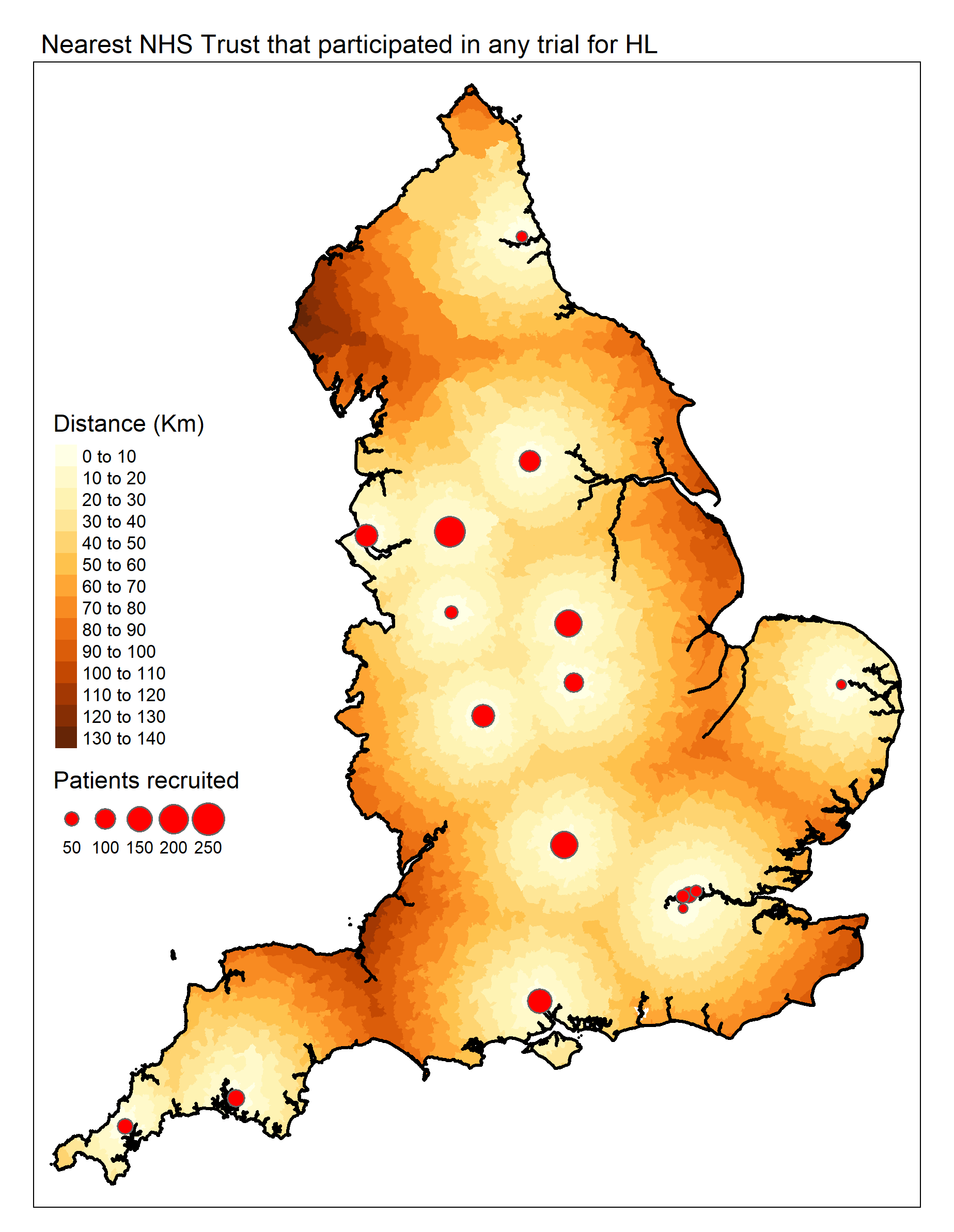
**

**Figures S5: Distance from each LSOA to nearest research active NHS Trust under definition 5): an NHS Trust that recruited to any FL clinical trial.**


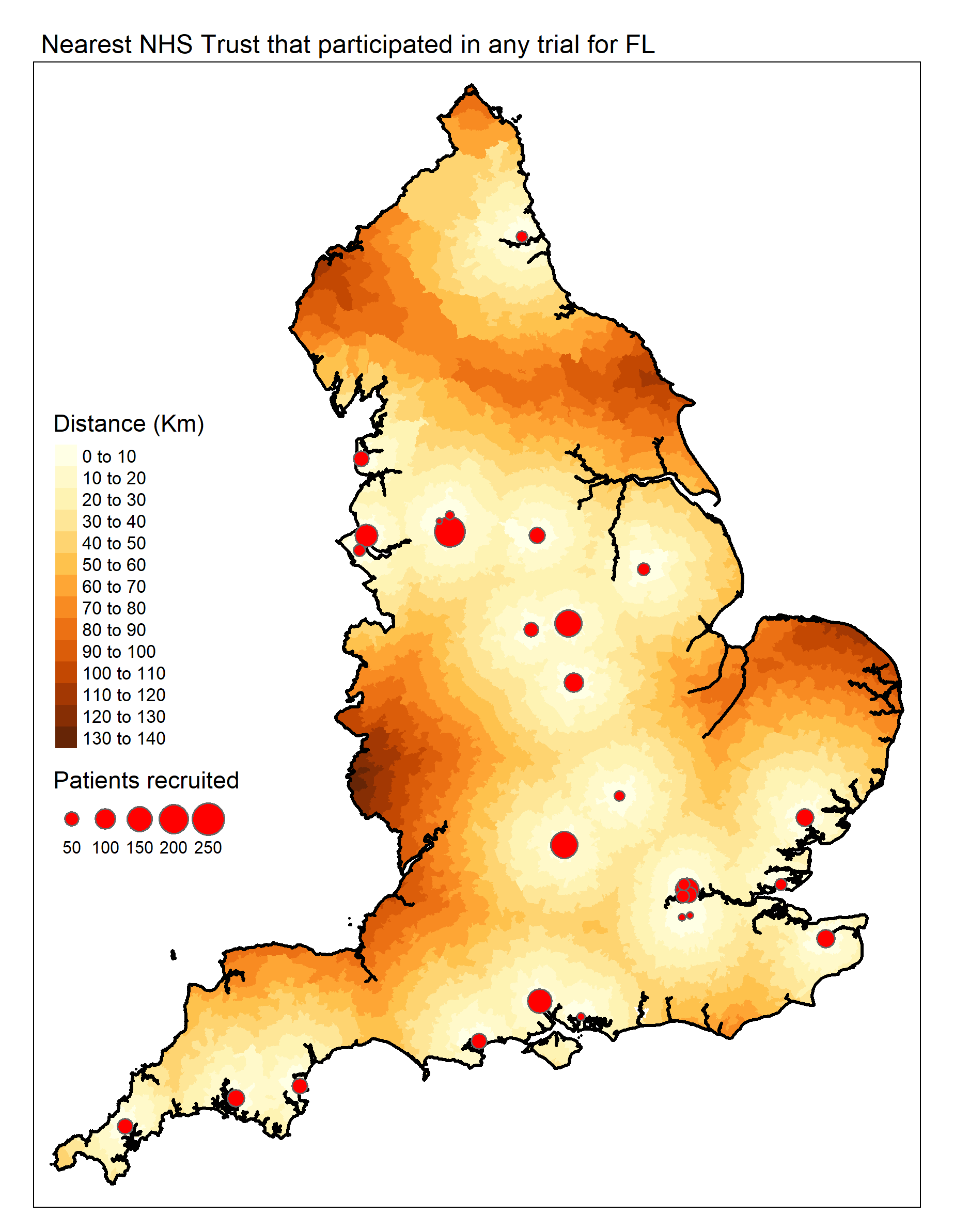

Supplement: Supplementary file 1 — Data S1. [file BJH-206-531-s002.docx]
